# Neural Network Evolving Algorithm Based on the Triplet Codon Encoding Method

**DOI:** 10.3390/genes9120626

**Published:** 2018-12-13

**Authors:** Xu Yang, Songgaojun Deng, Mengyao Ji, Jinfeng Zhao, Wenhao Zheng

**Affiliations:** School of Computer Science and Technology, Beijing Institute of Technology, Beijing 100081, China; songgaojun.deng@gmail.com (S.D.); 3220180810@bit.edu.cn (M.J.); 3220180901@bit.edu.cn (J.Z.); 3220180907@bit.edu.cn (W.Z.)

**Keywords:** DNA, neural evolution, triplet codon, encoding

## Abstract

Artificial intelligence research received more and more attention nowadays. Neural Evolution (NE) is one very important branch of AI, which waves the power of evolutionary algorithms to generate Artificial Neural Networks (ANNs). How to use the evolutionary advantages of network topology and weights to solve the application of Artificial Neural Networks is the main problem in the field of NE. In this paper, a novel DNA encoding method based on the triple codon is proposed. Additionally, a NE algorithm Triplet Codon Encoding Neural Network Evolving Algorithm (TCENNE) based on this encoding method is presented to verify the rationality and validity of the coding design. The results show that TCENNE is very effective and more robust than NE algorithms, due to the coding design. Also, it is shown that it can realize the co-evolution of network topology and weights and outperform other neural evolution systems in challenging reinforcement learning tasks.

## 1. Introduction

The birth of the computer made people’s lives more convenient. Research in the computer field has also promoted the production of more advanced technology, which will improve people’s future lives to a greater extent. At the end of the 20th century, Evolutionary Computing [1] and Artificial Neural Networks, as two branches of Artificial Intelligence (AI), merged and produced a new field, Neuroevolution (NE).

In nature, biological neural networks have evolved over a long period of time. After countless evolution and elimination processes, the biological neural network has produced the best individual characteristics to adapt to the present natural environment, and with the continuous changes in the natural environment, this evolutionary process will continue. As a subfield of AI, NE imitates the process of natural evolution using evolutionary algorithms to generate Artificial Neural Networks (ANNs). It is widely used in areas such as Artificial Life, General Game Playing and Evolutionary Robotics [2].

Basically, the learning algorithms of Artificial Neural Networks can be divided into supervised learning algorithms, semi-supervised learning algorithms, and unsupervised learning algorithms [3,4,5]. Among them, supervised learning is the most important and widely used machine learning algorithm in Artificial Intelligence. It uses the known data samples to train the algorithm model so as to achieve the required performance [6,7], and it has led to remarkable achievements in many fields.

However, supervised learning algorithms have specific requirements for inputs and outputs, and it often performs poorly when faced with the problems of the scarcity of known samples or the difficulty in collecting samples. In addition, the design of the model often faces the problems of parameter optimization and feature processing. Also, supervised learning algorithms often need to be trained many times (possibly millions), and need to consume huge computing resources, which is not an efficient solution. In contrast, NE only needs to measure the performance of the neural network on a task, greatly simplifying the problem and providing an efficient solution to the problem. NE can effectively combine the adaptive mechanism of evolutionary computing and the learning mechanism of the neural network to overcome many shortcomings of the traditional Artificial Intelligence learning model. Therefore, it is a promising research model.

At present, there are many neural evolutionary algorithms, which are mainly divided into two kinds [8]:

1. The traditional NE algorithm, which only evolves the connection weights of fixed network topology;
2. The Topology and Weight Evolving Artificial Neural Network (TWEANN). This algorithm can evolve the topology structure and connection weight of the network at the same time.

The development of the mainstream Deep Learning Neural Network (DNN) is based on the Artificial Neural Network (ANN) which was born decades ago. To some extent, it is similar to the brain. One of the key structures of the brain is the neuron, a small cell that sends signals to other neurons by connecting. When many neurons are connected to each other in networks, they become what we call neural networks. The research of ANN simulates the behavior of biological neurons and their signal propagation by programming [9].

In the study of ANN, neurons are often connected according to artificial rules, and, on this basis, the optimal weights are generated through algorithm training. Yet, brain behavior is determined not only by the weight of connections, but also by the overall structure of the brain itself. The existing learning algorithms of ANN can help to solve the weight problem, but where does the structure come from? From the point of view of natural evolution, the structure of a neural network evolves through a long history, that is, the evolution of a neural network structure is of great significance.

The study of NE has been developed for decades and has been greatly promoted. At present, the research focus of NE is the design of network encoding. The encoding methods of the neural network can be divided into the direct encoding method and the indirect encoding method.

The two encoding methods have their own pros and cons. Direct encoding does not need to consider the close relationship between gene composition and individual performance, but only needs to prove that its coding design can promote the effective evolution of the network. However, indirect encoding requires the design of a set of coding and decoding rules for the transformation of gene sequences and individual phenotypes, so it is necessary to understand more about the genetic and evolutionary mechanisms of biology.

The gene encoding mode of organisms is extremely efficient, and a very short gene sequence can control the complex phenotypes of individuals. Many of the current studies of indirect encoding are at the exploratory stage, but with the continuous development of the research on the mechanisms of biological evolution, research on the NE algorithm based on indirect encoding still has great potential.

In this paper, we propose an indirect encoding method that is suitable for NE, which encodes both the network structure and weights of network connections. Based on the proposed encoding and decoding method, we can transform gene sequences to network representation and can efficiently evolve the effective network of the target problem with the NE algorithm. This provides a reference value for the research of NE and the encoding of ANN for future research.

The following is organized as follows: A literature review is given in Section 2. Section 3 discusses some basic concepts and our design motive. The encoding method is presented in Section 4. In Section 5 and Section 6, two different encoding strategies are discussed in detail. The Triplet Codon Encoding Neural Network Evolving Algorithm (TCENNE) is proposed in Section 7. Experiments are shown in Section 8. Finally, conclusions are given in Section 9.

## 2. Literature Review

The encoding methods of the neural network can be divided into the direct encoding method and the indirect encoding method.

### 2.1. Direct Neural Network Encoding Methods

Direct encoding is an encoding strategy that directly encodes the parameters of neural networks, such as weights and connection information, into the genome. There are many different direct encoding methods:

#### 2.1.1. Connection-Based Direct Encoding Strategy

**Figure 1:**
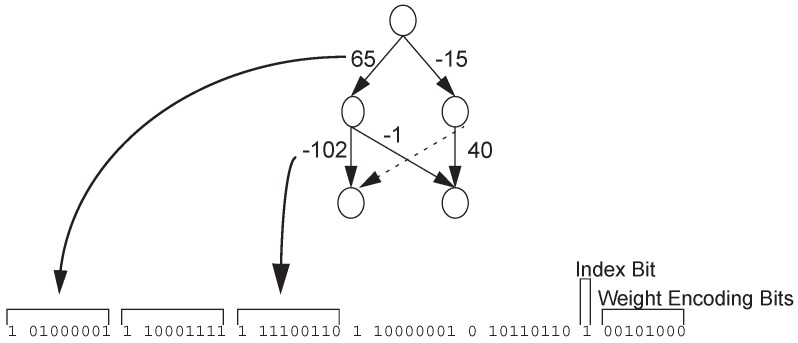
GENITOR encoding.

Most direct encoding strategies of neural networks are connection-based, that is, they encode and optimize connections on a predetermined network structure. Geoffrey et al. proposed a NE system called Innervator [10], in which each connection corresponds to one bit encoding. Each individual of each generation is trained several times to evaluate fitness. Experiments showed that the design has the ability to solve the problem and can converge quickly. Whitley et al. [11] presented the GENITOR encoding method, which encodes the weights of a given hierarchical network topology using bit strings, as shown in Figure 1 [11]. The Index Bit is used to indicate whether a connection exists or not. Weight Encoding Bits encode weights in binary form. Montana et al. proposed a encoding strategy similar to GENITOR [12,13], which encodes weights in real numbers, and some mutation operators suitable for the encoding are also defined. This encoding strategy can also be successfully applied in classification tasks. Maniezzo proposed an interesting improvement to Genitor encoding [14,15], where the evolution of granularity is introduced into the encoding method. He believes that the weights in binary encoding form make a bit string that is too long to evolve effectively in genetic algorithms. He encodes the bits of each weight in the parameter, which enables the algorithm to find the topology effectively with a small number of bits.

#### 2.1.2. Node-Based Direct Encoding Strategy

**Figure 2:**
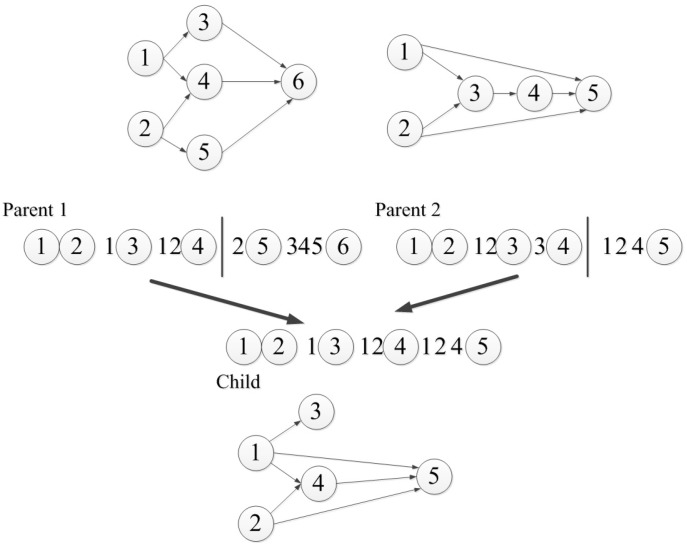
Schiffmann crossover operator.

**Figure 3:**
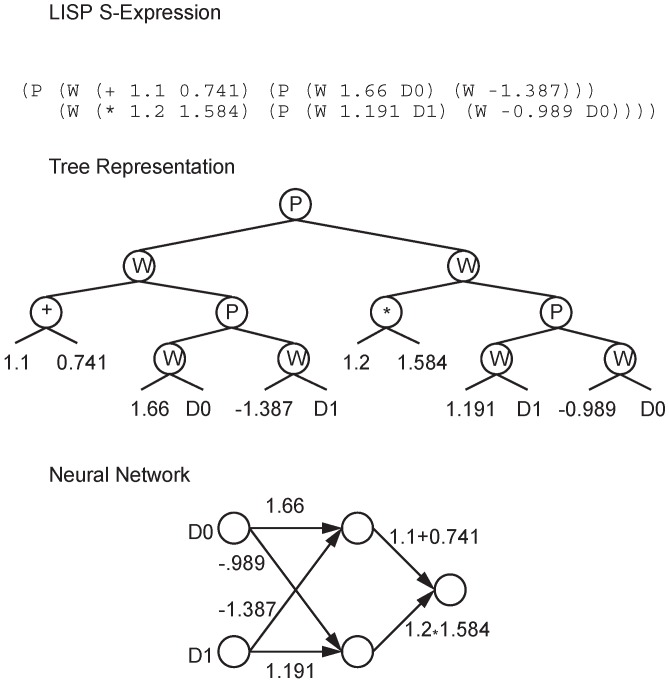
Koza encoding example.

The biggest drawback of the connection-based encoding strategy is that a basic network structure must be designed in advance, so the number of neurons is difficult to control. The node-based encoding strategy can solve this problem. In node-based encoding design, the parameter string is no longer composed of weights, but contains information about the whole node. Schiffmann et al. proposed a simple node-based encoding strategy [16], where the parameter string is a list of nodes that contain connection information. The crossover operator of their method is shown in Figure 2, which can only be set between nodes, and does not divide the existing connections. They applied their method to the application of thyroid data classification. White proposed a node-based encoding method called the GANNET hierarchical network [17], whose parameter string is a list of neurons with connections and location information. However, the structure of the neural network is limited in some aspects. The number of input connections per node is limited to four, and these are only between adjacent layers. Koza et al. proposed a method to apply genetic programming to the neural network through node-based encoding [18], where nodes are not encoded in parameter strings, but in parameter trees, as shown in Figure 3, where *P* represents a node, while *W* is the weight value. Neurons can have any number of subtrees with weights. A weight value has two subtrees: one contains the weight value and the other is the source of the connection, which must be a processing unit or an input node.

#### 2.1.3. Layer-Based Direct Encoding Strategy

**Figure 4:**
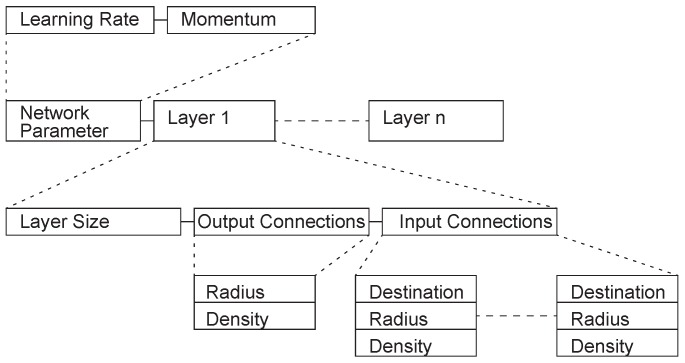
Mandischer encoding example.

Harp proposed a layer-based encoding scheme [19] in which the genome consists of a certain number of regions, each of which encodes a layer of the network. This encoding method can be successfully applied to the problem of XOR and 4x8 digit number recognition. Mandischer proposed an encoding method [20], whose basic structure is a list of network parameters and layers (Figure 4) but the weights are not encoded. Information about the size of each layer of the network and the input/output connections is stored. The system designed based on this encoding method can be used for quite complex tasks, such as the detection of edges/corners in 9 × 9 pixel images, the recognition of letters in 8 × 11 pixel images, and the Mexican hat function estimation.

#### 2.1.4. Route-Based Direct Encoding Strategy

**Figure 5:**
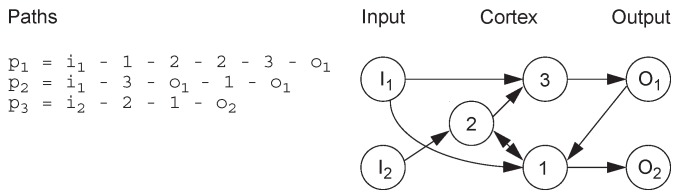
Route-based encoding example.

Jacob proposed a method encoding strategy based on routes in the network [21]. A route is defined as a list of neurons starting with input neurons and ending with output neurons, with no restrictions on the order of nodes in the route (Figure 5). Therefore, this encoding strategy does not necessarily generate feed-forward networks. The network generated by this encoding method can be applied to control tasks, such as ball balance.

### 2.2. Neural Network Indirect Encoding Methods

The direct encoding methods usually encode parameters such as the connections or weights in the genome, while in the indirect encoding, the encoding is not the parameter itself, but the production rule, which defines how these parameters are generated. This is a common biological status. Boers pointed out that the tissues of biological organisms exhibit great modularity, for example, the skin of the whole body is made up of the same types of skin cells. Therefore, by using certain rules, the encoding strategy of replicating units with the same function is more efficient in generating large networks.

**Figure 6:**
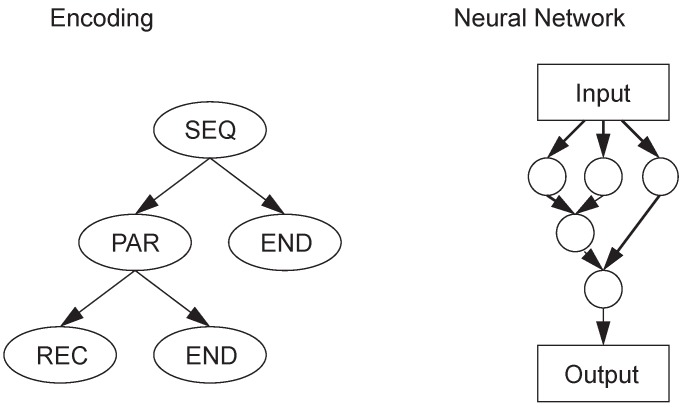
Cellular encoding example.

Kitano proposed the first indirect encoding scheme, called syntactic encoding [22], which has good scalability, although it is only used for simple encoding and decoding rules. Frederic Gruau proposed a cellular encoding method [23], as shown in Figure 6, which is represented by a tree structure. Aristid Lindemayer proposed a biological model called the Lindenmayer system [24], which attempts to mimic the cellular development of organisms, where cells exchange information only with neighbors. Strings representing the network are generated from a set of generation rules and the initial string. This encoding method can solve the problem of XOR and the recognition of simple letters.

## 3. Basic Concepts and Motive

### 3.1. Basic Concept of the Triplet Codon

In biology, DNA strands are the sequences of nucleotide bases, each of which consists of several nucleotide bases. There are four different forms of nucleotide base: adenine (A), guanine (G), cytosine (C) and thymidine (T).

**Figure 7:**
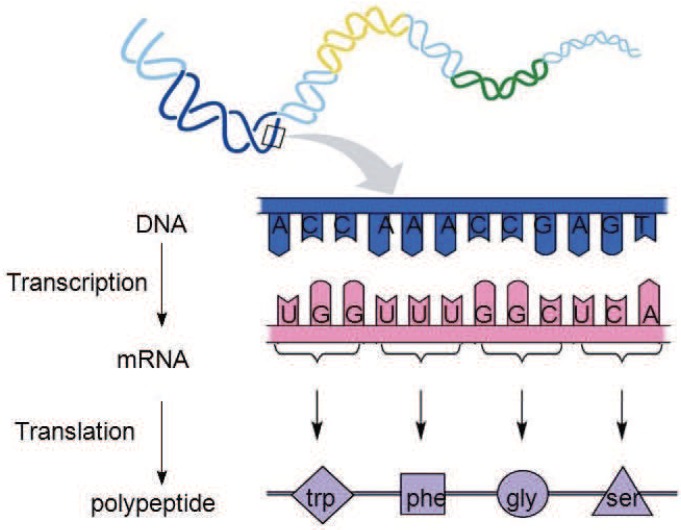
The translation process of DNA.

During cell reproduction, single strands of DNA are first translated into RNA sequences. RNA, like DNA, is a chain molecule formed by the condensation of ribonucleotides through phosphate bonds. In the process of DNA being translated into RNA, uracil (U) replaces thymidine (T) in DNA and becomes the characteristic base of RNA. RNA is then translated into amino acid sequences to form proteins [25], as shown in Figure 7.

DNA encodes the rules for the construction and growth of organs and tissues in organisms, which is an indirect encoding method. As can be seen from Figure 7, in a DNA single strand, the subsequences of three consecutive nucleotide bases are translated into one amino acid. The sequence of such three consecutive nucleotide bases is called a triplet codon, or a triplet for short.

The mapping relationship of the triplet nucleotide and amino acids is shown in Table 1 [26,27]. There are four different nucleotide bases in the DNA strands, namely, A G, C, and T, so there are 64 different combinations of the three consecutive nucleotide bases, but it can be seen from Table 1 that the 64 different triplet codons only correspond to 21 different amino acids. Therefore, in the process of translating DNA into amino acids, different triplet codons can be translated into the same amino acid, for example, TTC and TTT are both translated into Phe.

It can also be observed from Table 1 that there are two types of special triplet codon. The triplet codon ATG is the start codon, while the triplet codons TAA, TAG, and TGA are the stop codons, which are used as the starting and stopping signals in the process of amino acid translation into proteins. Therefore, there are only 19 amino acids expressed in biological DNA sequences.

This is actually a very beneficial feature. In the process of DNA mutation, there is diversity and redundancy in coding due to the existence of the many-to-one mapping relationship between triplet codons and amino acids in DNA. Thus, the transformation of a nucleotide base does not necessarily lead to a change in the translated amino acid, although it may result in a change in the triad codon. Thus, the accidental substitution of nucleotide bases can avoid the translation errors of amino acid sequences, so it will not cause a change in biological characters. To a certain extent, this property ensures the stability of genetic information transmission between generations.

### 3.2. Motive

More and more researchers are becoming interested in simulating the DNA genetic mechanism in computation [28,29,30,31,32], and in the field of neural evolution, a variety of network encoding strategies have emerged. The direct encoding method has been able to realize the common evolution of network topology and weights, but the existing research results of indirect encoding are still limited by the theoretical basis, and the development is not mature. From a biological point of view, DNA coding is a potential research direction. A network can be represented by a DNA strand, which can realize the diversity of DNA strand phenotypes by referring to the recombination and mutation of DNA in organisms, but, at the same time, it can guarantee a certain degree of reliability and stability.

So, in this paper, a triplet codon based DNA encoding method for neural network is proposed based on the process of DNA being translated into amino acids (omitting the part where DNA will be translated into RNA first). We propose the concept of the “component”. A neural network is presented by a single DNA strand, which consists of several components. Each component is represented by several triplet codons, and nodes, connections, and weight information are encoded in the component structure. By designing appropriate encoding and decoding strategies, the DNA strand can be decoded to form a complete neural network, and then optimized by the genetic evolutionary algorithm, and applied to many kinds of problem.

## 4. Triplet Codon Encoding Method

### 4.1. Overview

**Figure 8:**
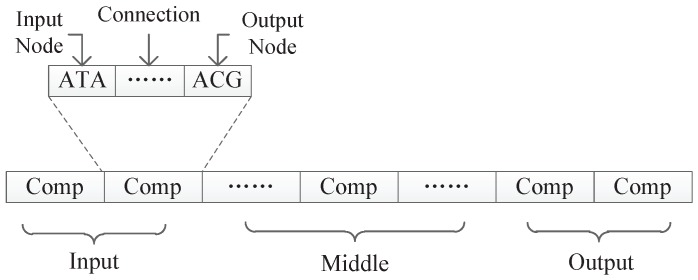
The format of a DNA strand.

In our proposed encoding method, a neural network is represented by a DNA strand, which consists of several components, as shown in Figure 8. Components are similar in structure, and each component is composed of several triplet codons. A triplet codon consists of three nucleotide bases, that is, A, G, C and T. Therefore, a DNA strand is actually composed of numerous nucleotide bases. The components in our method can be divided into three categories:

1. Input components: used to encode the input layer of the neural network;
2. Middle components: used to encode the hidden layer of the neural network, could be connected to the input layer or the output layer;
3. Output components: used to encode the output layer of the neural network.

### 4.2. Mechanism to Represent the Node Distribution of the Neural Network

Here are some basic definitions:

1. $N_{c}$: the number of components in a DNA strand, which is used to roughly determine the size of the network;
2. $N_{i}$: the number of input components, which is equal to the number of nodes in the input layer of the neural network;
3. $N_{o}$: the number of output components, which is equal to the number of nodes in the output layer of the neural network;
4. $N_{m}$: the number of middle components;
5. $n_{i}$: the number of nodes in the input layer of the neural network, or the input nodes of the neural network;
6. $n_{o}$: the number of nodes in the output layer of the neural network, or the output nodes of the neural network;
7. $n_{m}$: the number of nodes in the hidden layer of the neural network.

According to the definitions, the number of middle components $N_{m}$ can be calculated as

(1) $$N_{m}=N_{c}-N_{i}-N_{o}.$$

Additionally, $n_{i}$ is equal to $N_{i}$, while $n_{o}$ is equal to $N_{o}$. $n_{m}$ is decided according to the following equation:

(2) $$n_{m}=\left\lfloor |\sqrt{N_{c}}-1|\right\rfloor .$$

**Figure 9:**
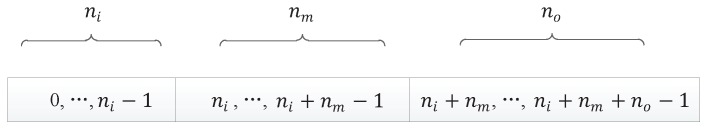
The node distribution in a neural network.

Actually, in our encoding method, the number of middle components is larger than the number of nodes in the hidden layer, which ensures the connectivity of the network. So, after calculation, we determine that the neural network has $n_{i}$ input nodes, $n_{m}$ hidden layer nodes, and $n_{o}$ output nodes. All nodes of the neural network are represented by a continuous natural number, as shown in Figure 9.

### 4.3. Mechanism to Interpret the Triplet Codon in the Component

As can be seen from Figure 8, the information contained in each component is similar and consists several triplet codons that are used to represent a component’s input node, output node, and connection information, respectively. In a component, the first triplet codon describes the input node of the component, the last triplet codon describes the output node of the component, and those several triplet codons in the middle can customize its rules. Two different encoding strategies are presented in this paper. In the first strategy, the connection information is represented as a subnet, while in the second strategy, the connection information is represented as the connection weight. Those two encoding strategies will be discussed in detain in the next section.

As mentioned above, there are 64 triplet codons, while 64 different triplet codons can be translated into 19 different amino acids. Referring to this mapping relation (shown in Table 1), we designed a rule to map the triplet codons in the component to integers 0 to 18, respectively—in total, 19 digits, as shown in Table 2. Since the start codon and stop codon cannot be translated into meaningful amino acids in the translation of DNA in organisms, their triplet codon and their adjacent triplet codon are translated into the same integer value in our encoding method. Thus, TAA, TAG, and TGA are translated as the same integer as TGG, that is, 9. Additionally, ATG is translated as the same integer as ATA, also 9.

By using this mapping method, all triplet codons in a DNA strand can be mapped to a unique integer.

## 5. Encoding Strategy 1—Connection Information Expressed as a Sub-Net Structure

Biological neural networks are extremely complex. However, DNA chains have a fixed length and contain a fixed amount of information, so how does DNA guide organisms to produce such complex neural networks? A reasonable conjecture is that there is a large amount of repetitive structural information in neural networks and there may be regulatory information in DNA to control the replication of some structures. If many types of subnets are defined, subnets will produce more complex networks in different connection combinations, and the information within the DNA strand will control the structure or location characteristics of those subnets.

**Figure 10:**
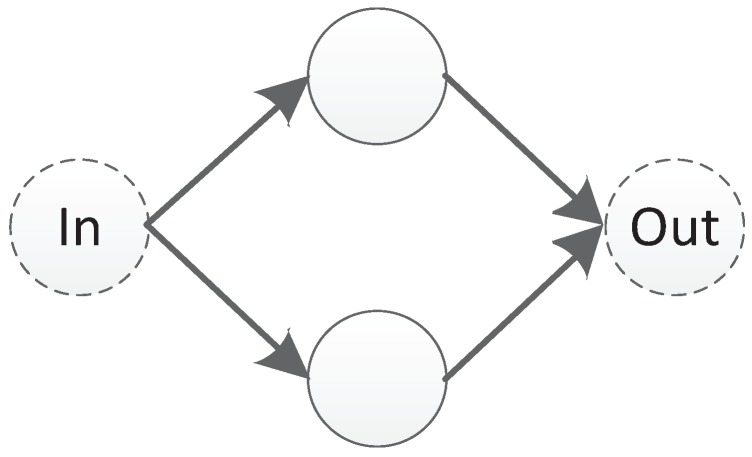
An example of a subnet.

In this paper, we propose a triplet codon-based DNA encoding strategy with a subnet structure. Under this encoding strategy, the connection information of a component is represented as a subnet. The so-called subnet is a predefined small scale network with input and output nodes, a small number of hidden nodes, and no connection weights, as shown in Figure 10. In Figure 10, a subnet with two hidden nodes is shown. The leftmost node is the input node and the rightmost node is the output node. The direction of the transmission of information follows the direction of the arrow, from left to right.

**Figure 11:**
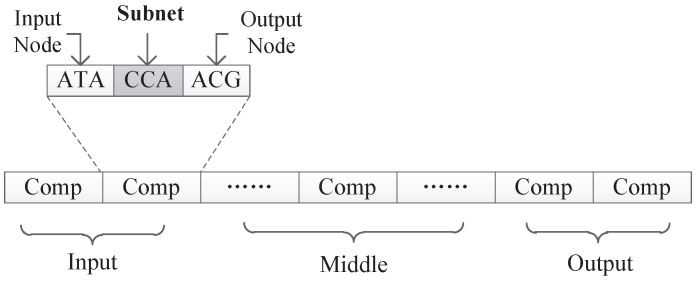
Format of components under the triplet codon-based DNA encoding strategy with the subnet structure.

Under this encoding strategy, each component is composed of exactly three triplet codons, as shown in Figure 11. The first triplet codon of a component describes the input node of that component. The third triplet codon of a component describes the output node of that component. Additionally, the second triplet codon of a component describes a kind of subnet structure associated with this component.

### 5.1. Mechanism to Interpret the First Triplet Codon of a Component

The first triplet codon in a component is used to represent the input node of that component. This triplet codon will first be mapped to an integer, and then mapped into the topology of the neural network.

#### 5.1.1. Input Component

The first $N_{i}$ components of a DNA strand are input components. Since input components are used to represent the $n_{i}$ (equal to $N_{i}$) input nodes of the neural network, the input node of an input component (described by the first triplet codon) can only be a node from the input layer of the neural network. Therefore, we need to map the first triplet codon of the $N_{i}$ input components to the $n_{i}$ input nodes of the neural network one-to-one. The algorithm to map the first triplet codon of an input component to a node in the neural network is shown in Algorithm 1.

**Algorithm 1** One-to-one mapping algorithm for the first triplet codon.**Input:**List of components *C*List of Nodes in the neural network $\left\{0,1,\ldots ,n_{i}-1\right\}$Number of candidate nodes *N***Output:**   List of mapped nodes *L*1:L=ϕ, $N=n_{i}$;2:**while** (C≠ϕ) **do**3: Pick a component *c* from *C*;4: Map the first triplet codon of *c* to integer *I*;5: **while** (ImodN)∈L **do**
6:  I=I+1;7: **end while**
8: Add *I* in *L*;9: Delete *c* from *C*;10:**end while**

**Figure 12:**
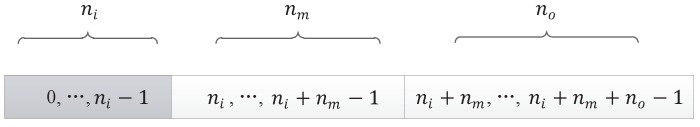
Valid list of nodes for the first triplet codon of the input component.

The first triplet codon of the input component can only be mapped to input nodes in the neural network, so the valid list of nodes in the neural network for the input component is $0,1,\ldots ,n_{i}-1$, as shown in grey in Figure 12.

#### 5.1.2. Middle Component or Output Component

**Figure 13:**
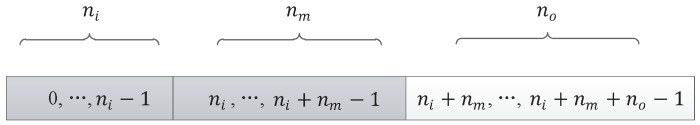
Valid list of nodes for the first triplet codon of a middle component or output component.

The input node of a middle component or an output component can be a node from the input layer or a node from a hidden layer, as shown in grey in Figure 13. The algorithm to map the first triplet codon of a middle component or an output component to a node in the neural network is shown in Algorithm 2.

**Algorithm 2** Regular mapping algorithm for the first triplet codon.**Input:**List of components *C*List of nodes in the neural network $\left\{0,1,\ldots ,n_{i}+n_{m}-1\right\}$Number of candidate nodes *N*
**Output:**   List of mapped nodes *L*1:L=ϕ, $N=n_{i}+n_{m}$;2:**while** (C≠ϕ) **do**3: Pick a component *c* from *C*;4: Map the first triplet codon of *c* to integer *I*;5: Add (ImodN) in *L*;6: Delete *c* from *C*;7:**end while**

### 5.2. Mechanism to Interpret the Last Triplet Codon of a Component

The last triplet codon in a component is used to represent the output node of that component. This triplet codon will first be mapped to an integer, then be mapped into the topology of the neural network.

#### 5.2.1. Input Component or Middle Component

**Figure 14:**
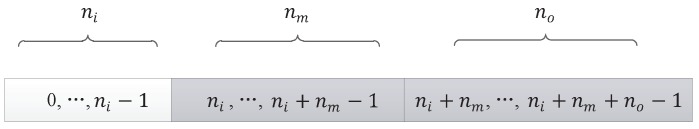
Valid list of nodes for the last triplet codon of input component or middle component.

The output node of an input component or a middle component could be node from hidden layer or node from output layer, as shown with grey color in Figure 14. We could use Algorithm 3 to map the last triplet codon of an input component or a middle component to a node in the neural network.

**Algorithm 3** Regular mapping algorithm for the last triplet codon.**Input:**List of components *C*List of nodes in the neural network $\left\{n_{i},n_{i}+1,\ldots ,n_{i}+n_{m}+n_{o}-1\right\}$Number of candidate nodes *N***Output:**   List of mapped nodes *L*1:L=ϕ, $N=n_{m}+n_{o}$;2:**while** (C≠ϕ) **do**3: Pick a component *c* from *C*, and map the last triplet codon of *c* to integer *I*;4: Add $\left(n_{i}+\left(I\;\mathrm{mod}\;N\right)\right)$ in *L*;5: Delete *c* from *C*;6:**end while**

#### 5.2.2. Output Component

**Figure 15:**
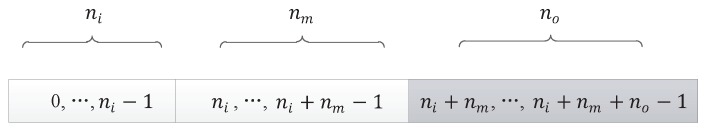
Valid list of nodes for the last triplet codon of the output component.

The output node of an output component can only be a node from the output layer of the neural network, as shown in grey in Figure 15. The last $N_{o}$ components of a DNA strand are output components. We need to map the last triplet codon of the $N_{o}$ output components to the $n_{o}$ (equal to $N_{o}$) output nodes of the neural network one-to-one. The algorithm used to map the last triplet codon of an output component to a node in the neural network is shown in Algorithm 4.

**Algorithm 4** One-to-one mapping algorithm for the last triplet codon.**Input:**List of components *C*List of nodes in the neural network $\left\{n_{i}+n_{m},n_{i}+n_{m}+1,\ldots ,n_{i}+n_{m}+n_{o}-1\right\}$Number of candidate nodes *N*
**Output:**   List of mapped nodes *L*1:L=ϕ, $N=n_{o}$;2:**while** (C≠ϕ) **do**3: Pick a component *c* from *C*, and map the last triplet codon of *c* to integer *I*;4: **while** (ImodN)∈L **do**
5:  I=I+1;6: **end while**7: Add $n_{i}+n_{m}+I$ in *L*;8: Delete *c* from *C*;9:**end while**

### 5.3. Mechanism to Interpret the Connection Information of a Component

**Figure 16:**
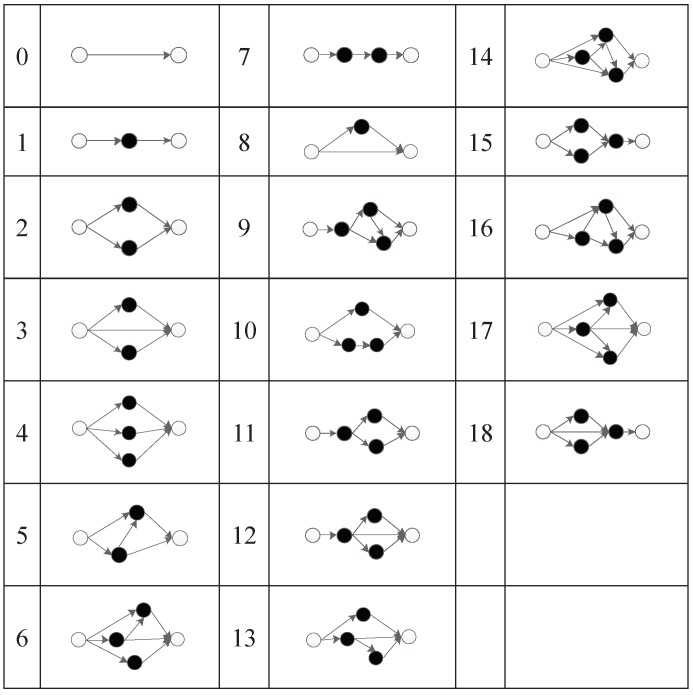
Mapping relationship between integers and subnet structures.

Under this encoding strategy, 19 subnets are set up. The subnet mapping relationship is shown in Figure 16. From the simplest direct connection subnet of non-hidden nodes to the complex connection subnet with three hidden nodes, each subnet has its corresponding serial number.

The second triplet codon of a component will first be mapped to an integer, and then mapped to the corresponding subnet structure. The subnet structure will be used to connect the input node of that component (the first triplet codon of that component) and the output node of that component (the third triplet codon of that component).

Under this encoding scheme, if both the input node and output node of a component in the DNA chain are found to be exactly the same as those of a component decoded before, then the representation of the newly decoded component is defined as implicit, and is not represented in the decoded neural network. Subsequent designs follow this rule.

### 5.4. An Example

Suppose we have a DNA strand with parameters set as $N_{c}=4,\;N_{i}=2,\;\mathrm{and}\;N_{o}=1$. Thus, $N_{m}$ is 1. Additionally, according to Equation (2), $n_{m}$ is 1. So, for this neural network, there are two nodes in the input layer, one node in the hidden layer, and one node in the output layer. The node set of this neural network can be expressed as {{0,1},{2},{3}}.

Suppose the DNA strand is

TAC CCT AGT CGT TTC AGC TCG CTA CCC TAG ACA CCG.

**Figure 17:**
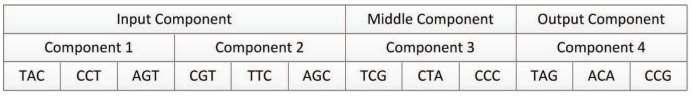
Example DNA strand exploiting encoding strategy 1.

As defined, there are two input components, one middle component, and one output component, as shown in Figure 17.

#### 5.4.1. Decoding of Component 1

Component 1 is an input component. Its first triplet codon is TAC, which represents component 1’s input node. According to Algorithm 1, TAC is first mapped to integer 3 (see Table 2). Since component 1 is an input component, its input node can only be mapped to nodes in the input layer, which are {0,1}. So, the number of candidate nodes *N* is 2. Because 3mod2 yields 1, the input node of component 1 is mapped to node {1} in the neural network.

Component 1’s output node can be mapped to nodes in the hidden or output layer that are {{2},{3}}, so the number of candidate nodes *N* is 2. The last triplet codon of component 1 is AGT, which is mapped to integer 2. According to Algorithm 3, 2mod2 yields 0, so the output node of component 1 is mapped to node {2} in the neural network.

**Figure 18:**
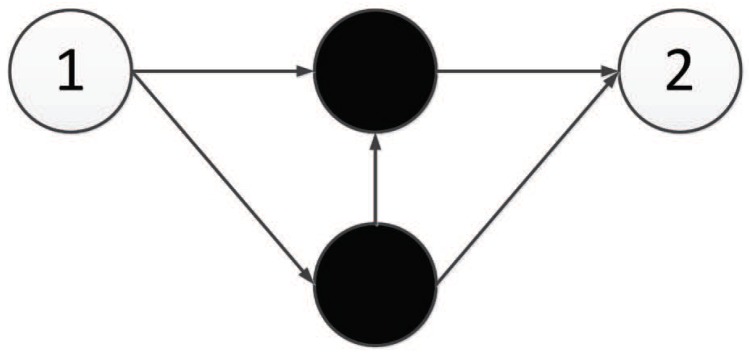
Subnet structure of component 1.

The second triplet codon of component 1 is CCT, which is mapped to integer 5. So, component 1 represents the structure shown in Figure 18.

#### 5.4.2. Decoding of Component 2

Component 2 is also an input component. Its first triplet codon is CGT. As with component 1, CGT is first mapped to integer 8, and then according to Algorithm 1, it is mapped to node {0} in the neural network. The last triplet codon is AGC, which can be mapped to integer 2, and according to Algorithm 3, mapped to node {2} in the neural network.

**Figure 19:**
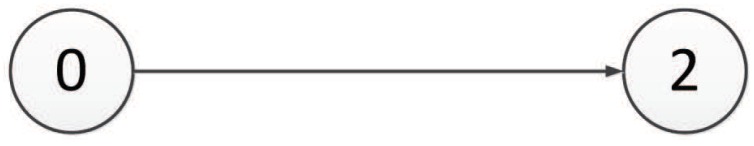
Subnet structure of component 2.

The second triplet codon is TTC, which is mapped to integer 0; thus, component 2 represents the structure shown in Figure 19.

#### 5.4.3. Decoding of Component 3

Component 3 is a middle component. Its first triplet codon is TCG. As a middle component, the input node of component 3 can be mapped to nodes in the input layer or hidden layer, that is {{0,1},{2}}. The number of candidate nodes *N* is 3. TCG is first mapped to integer 2, and according to Algorithm 2, mapped to node {2} in the neural network.

The last triplet codon is CCC. The output node of a middle component can be nodes in the hidden layer or output layer that are {{2},{3}}. CCC is first mapped to integer 5, and then according to Algorithm 3, mapped to node {3} in the neural network.

**Figure 20:**
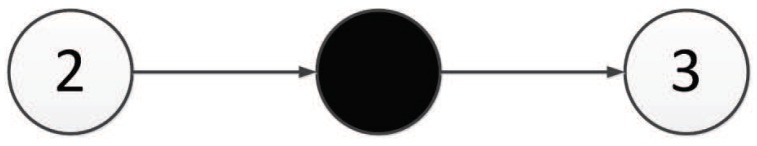
Subnet structure of component 3.

The second triplet codon is CTA, which is mapped to integer 1; thus, component 3 represents the structure shown in Figure 20.

#### 5.4.4. Decoding of Component 4

Component 4 is an output component. Its first triplet codon is TAG. As an output component, the input node of component 4 can be mapped to nodes in the input layer or hidden layer, that is {{0,1},{2}}. The number of candidate nodes *N* is 3. TAG is first mapped to integer 9, and then according to Algorithm 2, mapped to node {0} in the neural network.

The last triplet codon is CCG. As an output component, the output node of this component can only be nodes in the output layer, that is, {3}. The number of candidate nodes *N* is 1. CCG is mapped to integer 5, and then according to Algorithm 4, mapped to node {3} in the neural network.

**Figure 21:**
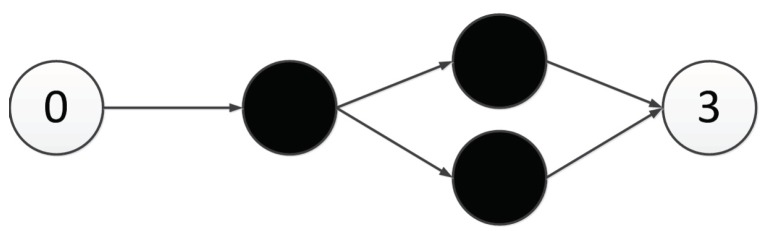
Subnet structure of component 4.

The second triplet codon is ACA, which is mapped to integer 11; thus, component 4 represents the structure shown in Figure 21.

#### 5.4.5. Integration Result of All Four Components

**Figure 22:**
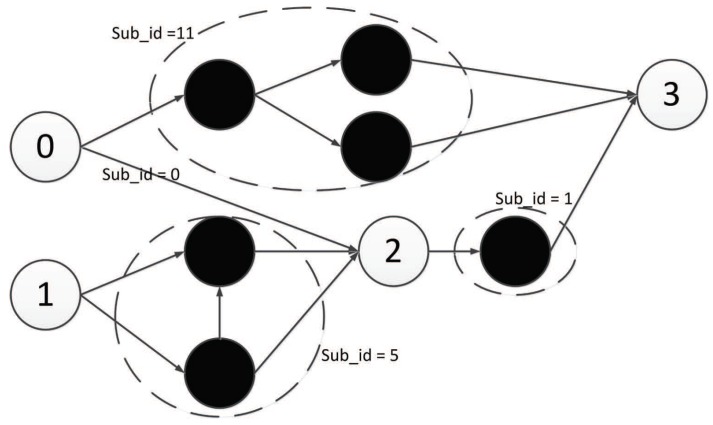
Whole structure of all four components.

So, after the integration of all four components, the whole structure of the neural network represented by this DNA strand is shown in Figure 22.

## 6. Encoding Strategy 2—Connection Information Expressed as Connection Weight

The encoding strategy with a subnet structure can only realize the evolution of the network topology without considering the importance of the connection weights in the current Artificial Neural Networks. If we need to adjust the weights, we need to design additional weight adjustment algorithms. Therefore, there are great limitations in the application of strategy 1.

**Figure 23:**
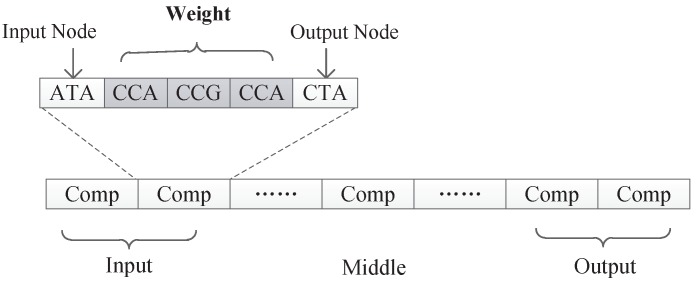
Format of DNA strand under encoding strategy 2.

In view of this, we propose a second encoding strategy. Under this encoding strategy, the connection information of a component is expressed as the connection weight. Because the weights are usually decimals in a certain range rather than integers, each weight variable is represented by three triplet codons to ensure that the decoded weights have a certain level of accuracy. Figure 23 shows the format of components under encoding strategy 2. Each component is composed of five triplet codons. The first triplet codon is used to represent the input node of that component, the last triplet codon is used to represent the output node of that component, and the middle three triplet codons are used to represent the connect weight.

Under encoding strategy 2, the input and output nodes of each component are interpreted in the same way as in encoding strategy 1, so we only focus on a discussion of the interpretation of the connection weight.

### 6.1. Mechanism to Interpret the Connect Information of a Component

In the encoding strategy 2, the weights are not random and have a definite range. First of all, we need to set the maximum and minimum values of weights to be $w_{max}$ and $w_{min}$, that is to say, the range of weights is [$w_{min}$, $w_{max}$]. Assuming that a nucleotide represents a unit length, the encoding length *l* of each weight can be expressed as l=3×k, where *k* represents the number of triplet codons in each weight encoding. Because, in the encoding strategy used in this paper, each weight is made up of three triplet codons, thus k=3. Each triplet codon can be mapped to an integer value according to Table 2, and *k* triplet codons can be mapped to a sequence of integers.

Assuming the middle three triplet codons in a component are mapped to an integer sequence as $\left\{bit_{1},bit_{2},bit_{3}\right\}$, the connection weight of that component *w* can be calculated as

(3) $$B=\sum_{j=1}^{k}\left(bit_{j}\right)\times 19^{l/3-j}$$

(4) $$w=\frac{B(w_{max}-w_{min})}{19^{l/3-1}}+w_{min}$$

where *B* is an intermediate variable, while Equation (4) is used to map the value of the intermediate variable *B* to weight *w* [32].

Suppose in one component, the connection weight is encoded as TTGGCAATA. Then, according to Table 2, those three triplet codons are mapped to the integer sequence {1,15,9}. Suppose the range of *w* should be [−10,10]. Then, according to Equations (3) and (4), the connection weight is w=−8.0898.

### 6.2. An Example

Suppose we have a DNA strand, with parameters set as $\left\{N_{c}=4,N_{i}=2,N_{o}=1\right\}$. Then, we can deduce that the number of nodes in the input layer $n_{i}$ (or the number of input components) is 2, and the number of nodes in the output layer $n_{o}$ (or the number of output components) is 1. According to Equation (2), the number of nodes in the hidden layer $n_{m}$ is 1. The node set of this neural network can be expressed as {{0,1},{2},{3}}.

Suppose the DNA strand is:

TAG TCT ATT GTA TTC GCT CTT CGG ATC GGC CCG TCC GAC GTT CAA ACC TAA TAC TGG TGC

**Figure 24:**
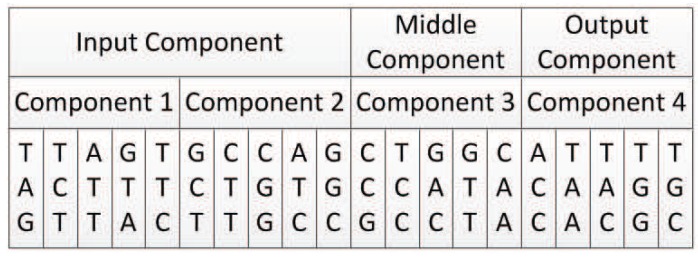
Example of DNA strand exploiting encoding strategy 2.

Then, there should be two input components, one middle component, and one output component. Additionally, each component is composed of five triplet codons, as shown in Figure 24.

#### 6.2.1. Decoding of Component 1

Component 1 is an input component. Its input node, which is expressed by its first triplet codon, can only be a node in the input layer of the neural network, so the candidate node list is {0,1}. Additionally, the number of candidate nodes *N* is 2. The first triplet codon of component 1 is TAG, which can be mapped to integer 9. According to Algorithm 1, the input node of component 1 is mapped to {1} of the neural network.

The output node of component 1 can be a node in the hidden layer or the output layer, which are {{2},{3}}. According to Algorithm 3, the last triplet codon of component 1 TTC is first mapped to integer 0, and then mapped to node {2} of the neural network.

Additionally, according to Equations (3) and (4), the middle three triplet codons which are used to represent connection weights are first mapped to an integer sequence, and then mapped to a connection weight as −3.650, as shown in Table 3.

#### 6.2.2. Decoding of Component 2

Component 2 is also an input component, the same as component 1, and the decoding results are shown in Table 4.

#### 6.2.3. Decoding of Component 3

Component 3 is a middle component. Its input nodes can be nodes in the input layer or hidden layer of the neural network, which are {{0,1},{2}}. Its output nodes could be nodes in the hidden layer or output layer of the neural network, which are {{2},{3}}. Its first triplet codon is CCG, according to Algorithm 2, which should be mapped to node {2} of the neural network. Its last triplet codon is CAA, according to Algorithm 3, which should be mapped to node {3} of the neural network.

Additionally, according to Equation (3) and Equation (4), the middle three triplet codons, which are used to represent connection weights, are first mapped to an integer sequence, and then mapped to connection weight as −3.484, as shown in Table 5.

#### 6.2.4. Decoding of Component 4

Component 4 is an output component. Its input nodes should be nodes in the input layer or hidden layer, that is, {{0,1},{2}}. Its first triplet codon is ACC, which can be mapped to integer 11, and according to Algorithm 2 mapped to node {2} of the neural network.

Its output node should be nodes in the output layer, that is, {3}. Its last triplet codon is mapped to this node according to Algorithm 4.

Additionally, according to Equations (3) and (4), the middle three triplet codons which are used to represent connection weights are first mapped to an integer sequence, and then mapped to a connection weight as −0.166, as shown in Table 6.

#### 6.2.5. Integration Result of All Four Components

**Figure 25:**
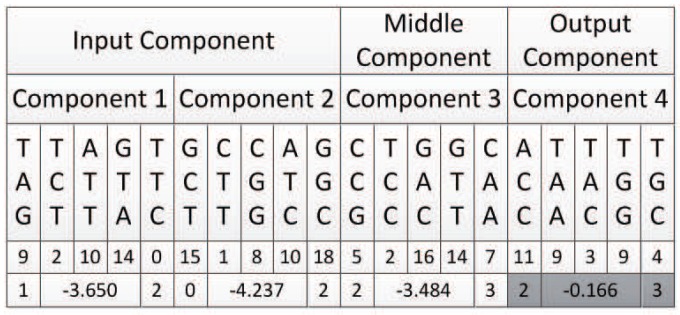
Decoding result of all four components.

The decoding results of all four components are shown in Figure 25. It should be mentioned that in this figure, the decoding results of component 4 are represented in grey. As discussed before, since the input and output nodes of component 4 are identical to those of component 3, component 4 is defined as implicit, meaning that it does not appear in the final neural network.

**Figure 26:**
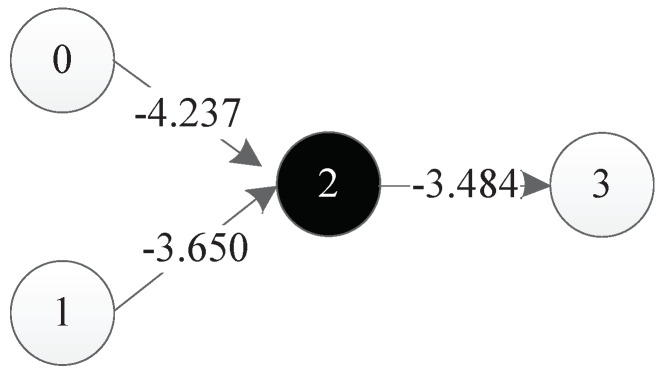
Integration result of all four components into the neural network.

So, the integration results of all four components of this DNA strand are shown in Figure 26.

## 7. Neural Network Evolving Algorithm Based on the Triplet Codon Encoding Method

**Figure 27:**
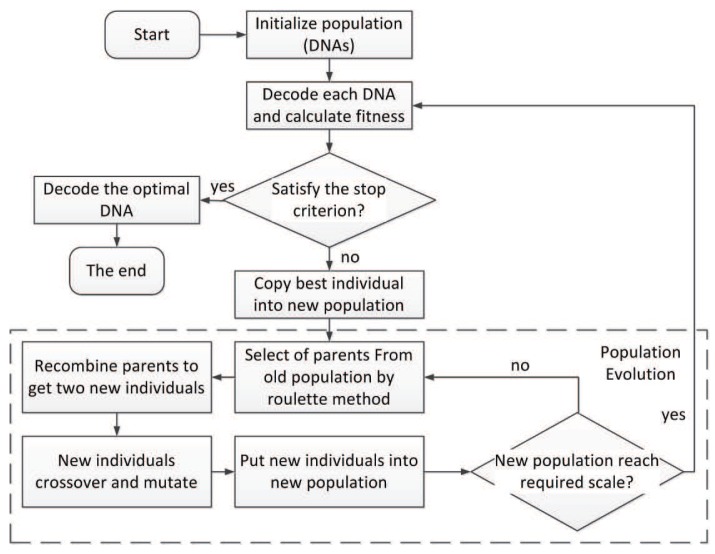
Flow of the Triplet Codon Encoding Neural Network Evolving Algorithm (TCENNE).

Based on the triplet codon encoding method proposed in the previous section, we present a neural network evolving algorithm called TCENNE. The flow of TCENNE is shown in Figure 27.

The basic idea of the algorithm is to search for the optimal individual in the neural network for the target problem by the genetic algorithm. In this algorithm, each individual is a DNA chain composed of multiple triplet codons. The DNA chain needs to be decoded into a neural network, then the fitness of the neural network will be calculated according to the requirements of the target problem. Specific genetic operators are designed for our encoding method.

### 7.1. Design of Genetic Operators

Three kinds of genetic operator were designed: crossover, mutation, and recombination.

#### 7.1.1. Crossover Operator

Single-point, multi-point, and arithmetic crossover operations have been used in existing genetic algorithms to mimic the process in which offspring inherit genetic information from their parents. Based on the triple codon encoding strategy proposed in this paper, three new crossover operators are defined. They are executed on the basis of a specified cross probability $P_{c}$. Each operator creates a new individual from an existing individual. These operators can easily be extended to DNA sequences of any length.

##### Move Operator

**Figure 28:**
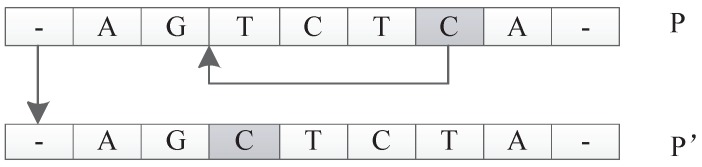
Move operator.

The input of this operator is an individual *P*. The function is to select a unit *u* in *P* randomly and relocate it to a new position to get a new individual $P^{\prime }$. For example, suppose individual *P* is represented as $u_{1}u_{2}u_{3}u_{4}u_{5}$. Here, each $u_{i}$ represents a unit in *P*. The move operator can generate a new individual $P^{\prime }$, which can be represented as $u_{1}u_{5}u_{2}u_{3}u_{4}$ by moving $u_{5}$ to the location following $u_{1}$. This operation can be extended arbitrarily because any unit can be moved to any new location. An illustration is shown in Figure 28.

##### Transform Operator

**Figure 29:**
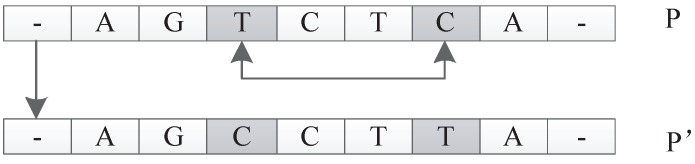
Transform operator.

The input of this operator is an individual *P*. The function is to generate a new individual $P^{\prime }$ by randomly exchanging two units’ positions of *P*. For example, suppose *P* is $u_{1}u_{2}u_{3}u_{4}u_{5}$. The transform operator could generate a new individual $P^{\prime }$ $u_{1}u_{4}u_{3}u_{2}u_{5}$ by randomly picking two units $u_{2}$ and $u_{4}$ and exchanging their positions. An illustration is shown in Figure 29.

##### Permutate Operator

**Figure 30:**
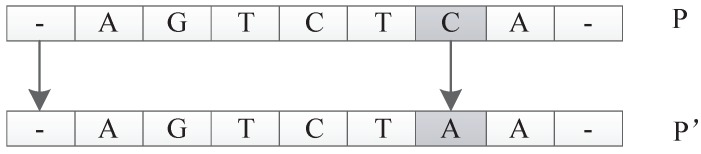
Permutate operator.

The input of this operator is an individual *P*. The function is to generate a new individual $P^{\prime }$ by random permutation of one unit of *P*. For example, suppose *P* is $u_{1}u_{2}u_{3}u_{4}u_{5}$. The permutate operator could randomly pick $u_{1}$ and replace $u_{1}$ with $u_{1}^{\prime }$ to get a new individual $P^{\prime }$ of $u_{1}^{\prime }u_{2}u_{3}u_{4}u_{5}$. An illustration is shown in Figure 30.

#### 7.1.2. Mutation Operator

In biology, mutation is a permanent change in the nucleotide sequence of an organism. Mutation operators are used to simulate DNA replicating mutations caused by mutagens such as chemical reagents and radiation. The mutation operator will change the structure of an individual randomly, increase the species diversity of a population, and prevent the algorithm from converging to the local optimum. In this paper, four mutation operators are introduced.

The first two are Reverse Mutations and Frequency Mutations, which are ordinary mutations and do not affect the length of DNA and are subject to probabilistic control of a parameter called $P_{m}$. The third is Adding Mutations, which can increase the length of DNA and are subjected to probabilistic control of a parameter called $P_{a}$. The fourth is Drop Mutations, which can shorten the length of DNA and are subjected to probabilistic control of a parameter called $P_{d}$. For our triplet codon encoding method, the length of a DNA strand can control the size of the neural network to a certain extent, that is, the more components in a DNA strand, the more hidden nodes and connections.

##### Reverse Mutation Operator

**Figure 31:**
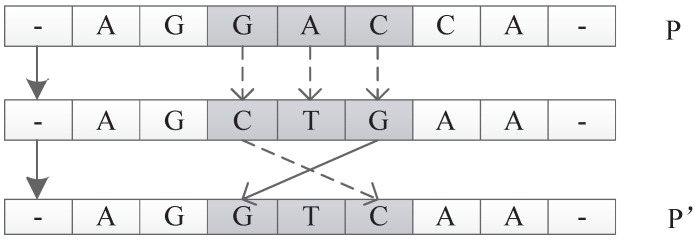
Reverse mutation operator.

The reverse mutation operates on the individual *P*. A triple codon unit of *P* is randomly selected, and its reversed triplet codon is used to replace the original triplet codon. Then, a new individual $P^{\prime }$ is returned. In biology, the anticodon of the codon is obtained by replacing each nucleotide with a complementary nucleotide according to the Watson–Crick complementarity principle. Therefore, A is replaced by T, and C is replaced by G, and vice versa. The reverse codon is obtained by reversing the nucleotide sequence of the anticodon. For example, for individual *P* shown in Figure 31, suppose we randomly pick the triplet codon GAC in *P*. The anticodon for GAC is CTG; thus, the reverse codon is GTC. So, the reverse mutation operator will replace GAC with GTC to generate a new individual $P^{\prime }$.

##### Frequency Mutation Operator

**Figure 32:**
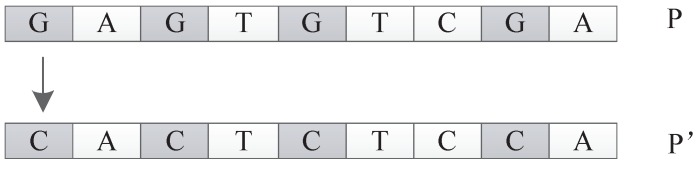
Frequency mutation operator.

The frequency mutation operator replaces the nucleotide with the highest frequency in the individual *P* with the nucleotide with the lowest frequency in the individual *P* and returns the new individual $P^{\prime }$. For example, as shown in Figure 32, in individual *P*, *G* is the nucleotide with the highest frequency, while *C* has the lowest frequency. The frequency mutation operator will replace all the *G* in *P* with *C* to generate a new individual $P^{\prime }$.

##### Adding Mutation Operator

**Figure 33:**
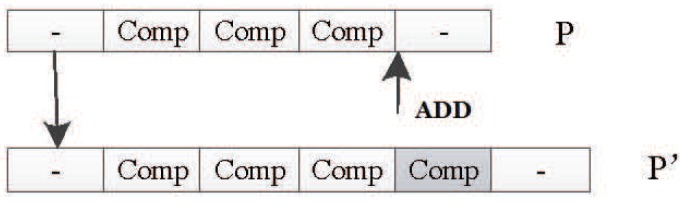
Adding mutation operator.

The polymorphism of the DNA fragment length in an organism is caused by the deletion, repetition, and insertion of a single nucleotide. In the encoding method designed in this paper, the DNA strand is composed of several components with the same structure. The components contain node and connection information. So, you can think of a component as a unit of DNA. The adding operator adds a unit randomly to the DNA of the input individual *P* to obtain a new and longer DNA chain $P^{\prime }$. Figure 33 shows how the adding mutation operator works by adding new units to the original DNA strand.

##### Drop Mutation Operator

**Figure 34:**
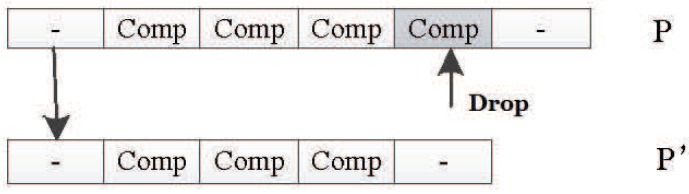
Drop mutation operator.

The drop mutation operator works like the adding mutation operator, and it can change the length of the DNA strand, too. It will randomly drop one unit in the original individual *P* to generate the new individual $P^{\prime }$. Figure 34 shows how it works.

#### 7.1.3. Recombination Operator

Genetic recombination is the process of producing offspring with different combinations of traits from any parent. In eukaryotes, gene recombination during meiosis produces a new set of genetic information that can be passed from the parent to the offspring. The recombination operation needs to be performed according to the predefined probability $P_{r}$. The recombination operator needs to perform two tasks:

1. According to the complementarities of nucleotides, two individuals of the parent generation are expanded from single-stranded DNA to double-stranded DNA, and the DNA sequence is cut by restriction enzymes.
2. The cut DNA fragment is recombined according to the complementarities to obtain the offspring individual.

The restriction enzyme cuts the DNA sequence and pastes the obtained fragments together on the premise that they have a matching sticky end. The recombination operation is based on the splicing model described by Amos and Paun [33].

### 7.2. Flow of the Algorithm

In the initialization phase of the algorithm, a population of a specified size is initialized. Each individual in the population is a chain of DNA composed of triplet codons. Then, each DNA strand is decoded into a neural network and the fitness is calculated. If there is a solution (an individual) to the target problem in the population, the DNA of the individual is decoded to obtain the target network and the algorithm is terminated.

If there is no solution, the individuals are sorted according to their fitness values, and the best individuals are first copied to the new population. This is an elite strategy in which all individuals with the highest fitness remain in the current group.

Then, a process is repeated to generate enough individuals for the new population from the old population. First, two parents were selected from the old population by roulette algorithm, and two offspring were obtained by recombination under a certain probability $P_{r}$. The offspring cross and mutate according to the set probability of a parameter called $P_{c}$, $P_{m}$, $P_{a}$, and $P_{d}$. The two newly created individuals are placed into the new population. This operation repeats until the population size reaches the specified value.

If the number of iterations of the program reaches the maximum number of iterations or the fitness of the optimal individuals in the current population meets the requirements of the problem, the algorithm will end. The algorithm can search the solution space iteratively to find the optimal global solution of a given problem.

## 8. Experiments

### 8.1. Experiment Setup

In this paper, we want to verify two hypotheses: (1) This encoding scheme can make the network evolve into a necessary structure; (2) compared with other neural evolutionary systems, this encoding can find a solution more effectively.

The XOR network construction experiment was chosen to verify hypothesis 1. This task is relatively simple, but it needs to add hidden neurons to the neural network. Therefore, this experiment can be used to test whether the proposed triplet codon encoding can evolve hidden neurons and to further verify whether the evolutionary algorithm based on this encoding method can properly evolve new structures to solve the target problem.

Double Pole Balancing with Velocity (DPV) and the more difficult Double Pole Balancing without Velocity without speed (DPNV) were chosen to verify hypothesis 2. The goal was to balance the two rods connected to the car by moving it in the right direction. Two-bar balance is a good benchmark experiment, and other neural evolution systems were tested in the experiment, so it is easy to compare the results of the algorithm. Among them, the task of balancing a two-pole car without speed is more difficult. This problem is a non-Markov chain task, so it is difficult to estimate the state of the next moment; thus, there is a higher requirement to keep the balance of the system. Therefore, this experiment can provide very powerful evidence for the effectiveness of the algorithm, that is, it can not only prove that the encoding strategy can improve the ability to find the optimal structure, but also is effective in very difficult control tasks.

The experiments conducted in this paper used the same parameters setting as in [34,35,36]. These three experiments can be solved by the reinforcement learning algorithm, but because the neural evolutionary algorithm can also solve this kind of problem very effectively, the neural evolution field usually regards it as the benchmark experiment.

All experiments in this paper adopted the encoding strategy with connection weights, because this encoding strategy can realize the co-evolution of weights and structures and there are suitable experimental data for comparison. The first encoding strategy with subnet structure can only evolve the network structure, and if the encoding method with connection weight proves to be effective, it must be able to prove that the first encoding can also maximize the evolution of the structure.

### 8.2. Results for the XOR Network Construction Experiment

**Figure 35:**
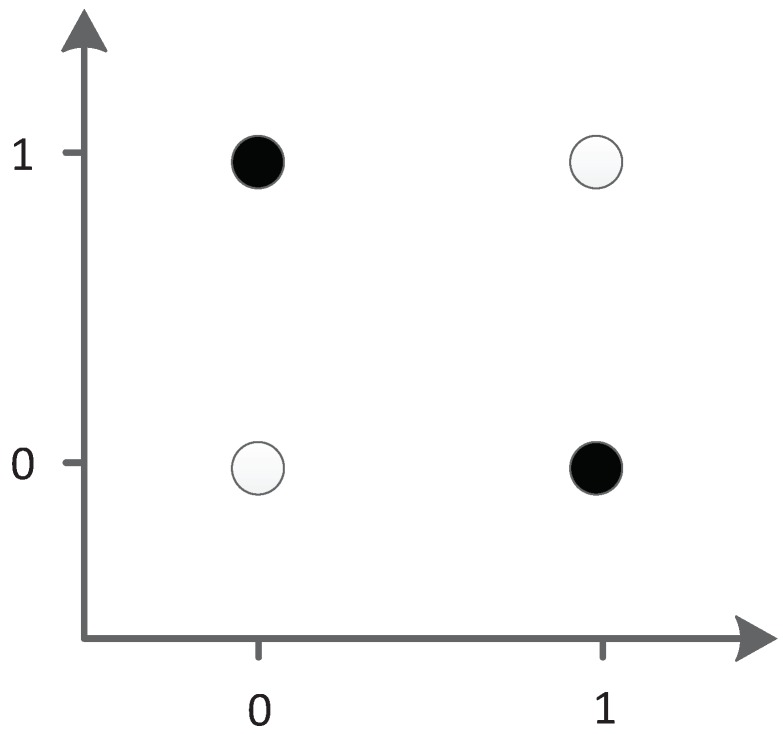
XOR problem.

The XOR problem is a typical linear inseparability problem, as shown in Figure 35, so it is necessary to add hidden neurons to solve the problem in neural networks. This requirement makes XOR suitable for testing the ability of algorithm structure evolution.

In the XOR experiment, the efficiency of the algorithm was compared by the average number of evaluation times. Table 7 compares different methods for a solution with the XOR network. Because this task was significantly easier to solve, we used a smaller population of 150. The results are averages over 100 runs.

The experimental results show that the structure of XOR was obtained by an average of 24 iterations in 100 XOR experiments, and the average number of evaluations was 3571 for our TCENNE algorithm. It can be seen from the data that the average number of times of evaluation and the number of iterations of the TCENNE algorithm were less than those of the NEAT algorithm with the same population size. Therefore, the TCENNE algorithm performs well in the simple neural network evolution problem.

In the 100 experiments completed, the network had 2.5 hidden nodes, on average. Additionally, there was no failure. The worst performance was 16,050 evaluation times, the population iteration was 107 times (average 23 generations), and all of the others were less than 10,000 evaluation times.

**Figure 36:**
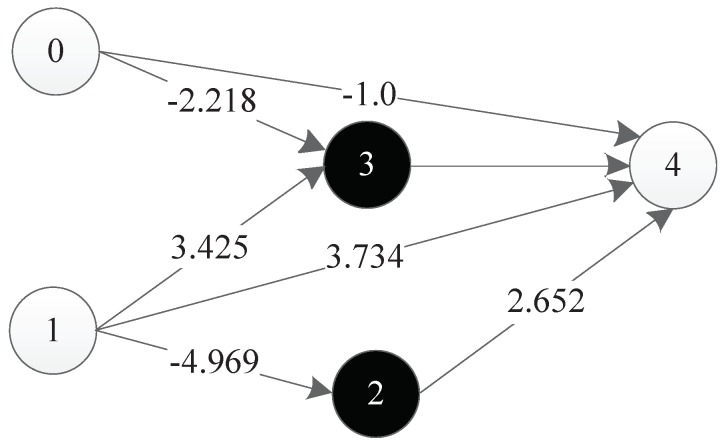
Neural network generated for the XOR problem.

The experimental results show that TCENNE can effectively use 1 to 3 hidden nodes to construct XOR networks. Figure 36 is a representation network of an optimal individual obtained from an experiment, where 0 and 1 represent input points, 4 represents output points, 2 and 3 represent hidden nodes obtained in the course of evolution, and each line has a corresponding weight.

The XOR problem has been used to prove the validity of neural network encoding and the performance of evolutionary neural network algorithm with topological weights. Although TCENNE can effectively solve the XOR problem, the design of the problem is too simple. In order to prove that TCENNE has the ability of continuous co-evolution of topology and weights, and that this triplet codon encoding method is actually beneficial to the evolution of network topology and weights, it needs to be verified for more difficult tasks.

### 8.3. Results for Double Pole Balancing with Velocity

**Figure 37:**
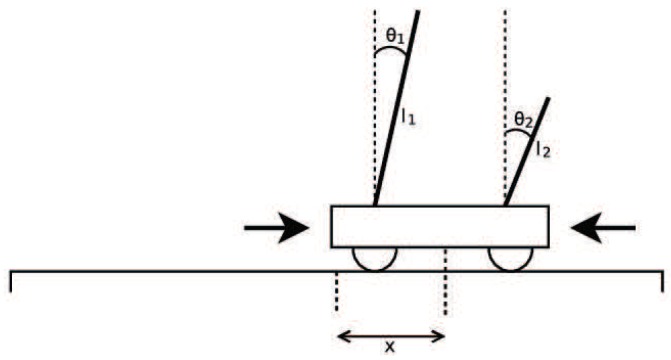
Illustration of two-bar balance problem.

Double Pole Balancing with Velocity (DPV) is a Markov problem. The experimental diagram is as shown in Figure 37. The standard of success of the task is to keep the system balanced by 100,000 time steps (30 minutes of simulation time). The vertical direction of the rod is considered to be balanced between $-36^{\circ }$ and $36^{\circ }$. The more time steps that the two bars keep in balance, the higher the fitness value is.

In the experiments, the number of individuals evaluated from the initial state to the optimal solution is represented by the number of evaluations. Iterations represent the algebra of population evolution in the algorithm. The population size represents the number of individuals in each generation. The relationship between the number of evaluations and the two can be expressed as

(5) (populationsize)×(iterations)≤(numberofevaluations).

Here, the relationship is smaller or equal, because in the last generation of populations, it is likely that the solution to the target problem can be found without having to evaluate all the individuals. In the experiment of DPV, if the evaluation time of the algorithm is smaller, the algorithm is more efficient.

In the DPV experiment, TCENNE was compared with five other published results of NE systems, as shown in Table 8.

For the statistical methods of the experimental data, the results of the first four systems are the average values of more than 50 experimental data. The results of the NEAT algorithm are the average values of more than 120 experimental data, while the results of the TCENNE algorithm are the average values of 242 experimental data. From the comparison of the experimental results, we can see that the experimental effect of the first three NE systems was much lower than that of the latter three.

Although there was no significant difference in the number of evaluations between the latter three neural evolutionary systems, and the number of TCENNE iterations was relatively high, TCENNE’s population size was set at 100 in the DPV experiment—less than the other two systems. Moreover, TCENNE was still superior to ESP and NEAT in terms of the evaluating time. Therefore, TCENNE is still competitive with the other two NE systems. Moreover, TCENNE selects the average value of more experiments, but the result was still better than the other two, which shows that this DNA encoding strategy based on triplet codon can ensure the stability and effectiveness of the algorithm.

In this encoding method, multiple codons can be decoded into the same amino acid, so the mutation of codons can keep the decoding invariance in a small range, and, at the same time, it keeps the diversity of the whole encoding. Because the mutation operation of DNA includes the adding mutation and the drop mutation, the network size will be different with the adding and drop mutation operation in the iterative process. As a result, smaller solutions can be found with the same performance.

Therefore, TCENNE can quickly find a small scale network to prove the effectiveness of the proposed encoding strategy.

### 8.4. Results for Double Pole Balancing without Velocity

Double Pole Balancing without Velocity (DPNV) is a non-Markov problem, that is, it is impossible to predict the state of the next moment through the historical state of the system. The input of the system is only the position of the car, the angle of the short rod and the angle of the long rod, so the system needs to keep its internal stability without speed. In the experiment, the TCENNE system needs to output a force acting on the car each time to control the direction and speed of the car and the double rods.

In 1996, Gruau et al. [41] introduced a special fitness function for this problem to prevent the system from simply moving the vehicle back and forth to keep the balance of the bar. This fitness evaluation method, which does not need to calculate the missing velocity value, has been used in many experiments of neural evolutionary systems. The fitness function *F* is composed of two subfitness functions, $f_{1}$ and $f_{2}$, and we can set $F=0.1f_{1}+0.9f_{2}$. Within 1000 time steps, the two subfitness functions are defined as

(6) $$f_{1}=\frac{t}{1000}$$

(7) $$f_{2}=\left\{\begin{array}{c} 0,\;if\;t<100\\ \frac{K}{\sum_{i=t-100}^{t}\left(\right|x^{i}\left|+\right|{\dot{x}}^{i}\left|+\right|\theta_{1}^{i}\left|+\right|{\dot{\theta }}_{1}^{i}\left|\right)},\;otherwise \end{array}\right.$$

where *t* is the number of time steps in which the two rods are balanced in 1000 total time steps. *K* is a constant which is set to 0.75. The denominator in Equation (7) is the sum of the absolute values of the state variables of the running car and the long rod. Summation involves the sum of the absolute values of state variables in the last 100 time steps of the run. Therefore, by minimizing damping oscillation, the system can maximize its fitness value.

According to Gruau’s solution standards, the best individuals of each generation need to perform generalization tests to ensure the robustness of their systems. Generalization testing takes more time than fitness testing, so it usually applies only to the best individuals of each generation. In the generalization test, in addition to requiring the two poles on the car to balance 100,000 time steps, the best individual network must balance the two poles in 625 different initial states. In each different initial state, success is achieved if the car has 1000 steps to keep both poles balanced. The total number of successes is the generalization performance of the solution. The system under test is generally required to succeed at least 200 times in 625 different states.

Therefore, there are two main criteria for evaluating DPNV: the number of evaluations and the number of generalizations. The number of evaluations is the number of individuals evaluated from the beginning to the end of the algorithm, and the number of generalizations is the number of times the optimal individual passes the generalization test. If the number of generalization is qualified, the smaller the number of evaluations is, the more efficient the algorithm is. If the number of generalizations is larger, the stability of the optimal individual is more stable, which indicates that the stability of the algorithm is stronger.

Since the non-Markov problem is more difficult than the Markov problem, the initial DNA chain should be set longer, which can make the variation of individual populations a little larger, so the initial component length $N_{c}$ of the DNA chain for our encoding method was set to 30.

Our proposed TCENNE algorithm was compared with five standard neuroevolutionary systems that can successfully solve DPNV problems. The experimental results are shown in Table 9.

The success of Cellular Encoding (CE) [41] is due first and foremost to the ability of its evolutionary structure. The fixed topology neural evolutionary system ESP can accomplish the task five times faster than CE by random startup of hidden layer nodes [40]. NEAT uses direct coding of the network topology and weight information and uses historical feature markers to make its structure evolve effectively [34]. AGE (Analog Genetic Encoding) [35] is a new implicit neural network evolutionary method based on the observation of biological genetic regulatory networks. The effect of this method on DPNV experiments is better than that of NEAT. The Echo State Network (ESN) is a kind of cyclic neural network based on a large number of sparse random connections. Unsupervised learning of ESN (UESN) applies the evolutionary continuous parameter optimization method to the evolutionary learning of ESN and verifies it effectively on the two-bar equilibrium system [36].

All of the algorithms in Table 9 use the same experimental settings, including the car, the parameters of the two rods, and the calculation method of the fitness function. The experimental results of TCENNE were taken as the average of 26 times, and the results of other algorithms were taken as the average of 20 times.

The average number of evaluations of the TCENNE algorithm was only 19074 times, which is only 1/44 of CE, which indicates that the improvement of structure evolution has great influence on the performance. This DNA encoding design based on the triplet codon can greatly improve the evolution efficiency of the structure. The average evaluation number of TCENNE was nearly 1/8 of that of ESP, which shows that the method of structure and weight evolution at the same time is better than that of fixed topology and evolution weight alone. The TCENNE algorithm was shown to be 1.7 times faster than NEAT and 1.2 times faster than UESN. Thus, the effectiveness of this encoding strategy is proved.

In the NEAT system, the connection and weight are coded directly, and the individual cross is realized by the unique mark of connection. The network structure of the population can evolve effectively in the iterative process. Although NEAT is a highly efficient neural evolutionary algorithm, it needs to encode a large amount of specific information to ensure the transmission of information between generations. The triplet codon based the encoding method designed in this paper adopts a more abstract encoding and decoding strategy. It can effectively evolve the individual structure and weight of the population by simply encoding a small amount of information.

In the TCNNE algorithm, different individuals can evolve into different structures, and each structure represents a different dimension space. Additionally, the simple genetic operator makes TCENNE try to solve the problem in many different ways. The change space is very large, and it is beneficial to the search of the optimal solution, which further reflects its advantages.

There were no significant differences in the performance of the previous four methods in the generalization test, but the average generalization time of the TCENNE experiment was 610, very close to the maximum 625, and far higher than the other four. Actually, in the 26 generalization experiments of TCENNE, only in one test was the number of generalizations 241, and the result for the others was 625. Thus, the optimal solution selected by the TCENNE algorithm can not only meet the requirements of the basic equilibrium time of the experiment, but can also maintain a balance of 1000 steps under most initial state conditions of the car systems. The stability of individuals also reflects the stability of the whole algorithm to a great extent.

TCENNE was determined to be the most efficient and more stable algorithm in these systems by comparing the number of evaluation times and the number of generalizations. This shows that the design of this triplet codon based coding is helpful for the co-evolution of weights and topologies. Additionally, it can make the remaining individual more stable.

## 9. Conclusions

In this paper, a novel DNA encoding method based on triplet codons and a neural evolutionary algorithm, TCENNE, was designed, and a set of genetic operators based on this encoding method was proposed. The performance of the TCENNE algorithm in experiments on the XOR network and two-bar balance was much better than that of other neural evolution systems, which verifies the rationality and validity of the coding design. Compared with the experimental results of other systems, the improvement of the efficiency of the algorithm is mainly due to the design of coding.

The improvement of the efficiency of the algorithm mainly lies in the coding design: (1) Nodes and connections are simply encoded in a DNA sequence consisting of four nucleotides, which solves the problems of crossover, recombination, and other genetic operations that are difficult to achieve; and (2) the introduction of the triplet codon and amino acid multiple-to-one transformation relationship in biological DNA can control the mutation of the network within a reasonable range, while ensuring the diversity of network topology and weights.

## Figures and Tables

**Table 1:** The mapping of DNA to amino acids.

1st Base	2nd Base								3rd Base
	T		C		A		G		
T	TTT	Phe	TCT	Ser	TAT	Tyr	TGT	Cys	T
	TTC		TCC		TAC		TGC		C
	TTA	Leu	TCA		$TAA^{\left[B\right]}$	Stop	$TGA^{\left[B\right]}$	Stop	A
	TTG		TCG		$TAG^{\left[B\right]}$		TGG	Trp	G
C	CTT		CCT	Pro	CAT	His	CGT	Arg	T
	CTC		CCC		CAC		CGC		C
	CTA		CCA		CAA	Gln	CGA		A
	CTG		CCG		CAG		CGG		G
A	ATT	Ile	ACT	Thr	AAT	Asn	AGT	Ser	T
	ATC		ACC		AAC		AGC		C
	ATA		ACA		AAA	Lys	AGA	Arg	A
	$ATG^{\left[A\right]}$	Met	ACG		AAG		AGG		G
G	GTT	Val	GCT	Ala	GAT	Asp	GGT	Gly	T
	GTC		GCC		GAC		GGC		C
	GTA		GCA		GAA	Glu	GGA		A
	GTG		GCG		GAG		GGG		G

**Table 2:** Mapping triplet codons to integers.

1st Base	2nd Base								3rd Base
	T		C		A		G		
T	TTT	Phe(0)	TCT	Ser(2)	TAT	Tyr(3)	TGT	Cys(4)	T
	TTC		TCC		TAC		TGC		C
	TTA	Leu(1)	TCA		$TAA^{\left[B\right]}$	Stop(9)	$TGA^{\left[B\right]}$	Stop(9)	A
	TTG		TCG		$TAG^{\left[B\right]}$		TGG	Trp(9)	G
C	CTT		CCT	Pro(5)	CAT	His(6)	CGT	Arg(8)	T
	CTC		CCC		CAC		CGC		C
	CTA		CCA		CAA	Gln(7)	CGA		A
	CTG		CCG		CAG		CGG		G
A	ATT	Ile(10)	ACT	Thr(11)	AAT	Asn(12)	AGT	Ser(2)	T
	ATC		ACC		AAC		AGC		C
	ATA	Met(9)	ACA		AAA	Lys(13)	AGA	Arg(8)	A
	$ATG^{\left[A\right]}$		ACG		AAG		AGG		G
G	GTT	Val(14)	GCT	Ala(15)	GAT	Asp(16)	GGT	Gly(18)	T
	GTC		GCC		GAC		GGC		C
	GTA		GCA		GAA	Glu(17)	GGA		A
	GTG		GCG		GAG		GGG		G

**Table 3:** Mapping result of component 1.

Component 1				
TAG	TCT	ATT	GTA	TTC
9	2	10	14	0
1	−3.650			2
Input Node	Connection Weight			Output Node

**Table 4:** Mapping result of component 2.

Component 2				
GCT	CTT	CGG	ATC	GGC
15	1	8	10	18
0	−4.237			2
Input Node	Connection Weight			Output Node

**Table 5:** Mapping result of component 3.

Component 3				
CCG	TCC	GAC	GTT	CAA
5	2	16	14	7
2	−3.484			3
Input Node	Connection Weight			Output Node

**Table 6:** Mapping result of component 4.

Component 4				
ACC	TAA	TAC	TGG	TGC
11	9	3	9	4
2	−0.166			3
Input Node	Connection Weight			Output Node

**Table 7:** Comparison for the XOR network experiment.

Method	Evaluations	Iterations	Population Size
NEAT [34]	**4755**	32	150
TCENNE	**3571**	24	150

**Table 8:** Comparison for the Double Pole Balancing with Velocity (DPV) experiment.

Method	Evaluations	Iterations	Population Size
Ev. Programming (2002) [37]	307,200	150	2048
Conventional NE (1991) [38]	80,000	800	100
SANE (1996) [39]	12,600	63	200
ESP (1999) [40]	3800	19	200
NEAT (2002) [34]	3600	24	150
TCENNE	3400	34	100

**Table 9:** Comparison for the Double Pole Balancing without Velocity (DPNV) experiment.

Method	Evaluations	Generalizations	Population Size
CE (1996) [41]	840,000	300	16,384
ESP (1999) [40]	169,466	289	1000
NEAT (2002) [34]	33,184	286	1000
AGE (2006) [35]	25,065	317	-
UESN (2008) [36]	23,571	241	-
TCENNE	19,074	610	150

## References

[1] Back T., Schwefel H. (1993). An overview of evolutionary algorithms for parameter optimization. Evol. Comput..

[2] Risi S., Togelius J. (2017). Neuroevolution in games: State of the art and open challenges. IEEE Trans. Comput. Intell. Ai Games.

[3] Bengio Y., Courville A., Vincent P. (2013). Representation learning: A review and new perspectives. IEEE Trans. Pattern Anal. Mach. Intell..

[4] Schmidhuber J. (2015). Deep learning in neural networks: An overview. Neural Netw..

[5] Lecun Y., Bengio Y., Hinton G. (2015). Deep learning. Nature.

[6] Norvig P. (2010). Artificial Intelligence: A Modern Approach.

[7] Schapire R., Freund Y. (2012). Foundations of Machine Learning.

[8] Togelius J., Schaul T., Schmidhuber J., Gomez F. (2008). Countering poisonous inputs with memetic neuroevolution. Parallel Problem Solving from Nature—PPSN X. PPSN 2008.

[9] Kleene S.C. (1951). Representation of events in nerve nets and finite automata. Autom. Stud..

[10] Miller G.F., Todd P.M., Hegde S.U. Designing neural networks using genetic algorithms. Proceedings of the International Conference on Genetic Algorithms.

[11] Whitley D., Starkweather T., Bogart C. (1990). Genetic algorithms and neural networks: Optimizing connections and connectivity. Parallel Comput..

[12] Montana D.J., Davis L. Training feedforward neural networks using genetic algorithms. Proceedings of the International Joint Conference on Artificial Intelligence.

[13] Montana D.J. (1991). Automated Parameter Tuning for Interpretation of Synthetic Images. Handbook for Genetic Algorithms.

[14] Maniezzo V. (1993). Searching among Search Spaces: Hastening the Genetic Evolution of Feedforward Neural Networks.

[15] Maniezzo V. (1994). Genetic evolution of the topology and weight distribution of neural networks. IEEE Trans. Neural Netw..

[16] Schiffmann W., Joost M., Werner R. Performance evaluation of evolutionarily created neural network topologies. Proceedings of the International Conference on Parallel Problem Solving from Nature.

[17] White D.W. (1993). GANNET: A Genetic Algorithm for Searching Topology and Weight Spaces in Neural Network Design. The First Step in Finding a Neural Network Solution.

[18] Koza J.R., Rice J.P. Genetic generation of both the weights and architecture for a neural network. Proceedings of the Ijcnn-91-Seattle International Joint Conference on Neural Networks.

[19] Harp S.A. Towards the genetic synthesis of neural networks. Proceedings of the Third International Conference on Genetic Algorithms.

[20] Mandischer M. (1993). Representation and Evolution of Neural Networks.

[21] Jacob C., Rehder J. (1993). Evolution of Neural Net Architectures by a Hierarchical Grammar-Based Genetic System.

[22] Kitano H. (1990). Designing neural networks using genetic algorithms with graph generation system. Complex Syst..

[23] Gruau F., L’universite C.B.I., Doctorat O.A.D.D., Demongeot M.J. (1994). Neural Network Synthesis Using Cellular Encoding and The Genetic Algorithm. Ph.D. Thesis.

[24] Lindenmayer A. (1968). Mathematical models for cellular interactions in development. J. Theor. Biol..

[25] Haselkorn R., Rothmandenes L.B. (1973). Protein synthesis. Annu. Rev. Biochem..

[26] Kimball J. (2010). The Genetic Code. Cold Spring Harb. Symp. Quant. Biol..

[27] Nakamoto T. (2009). Evolution and the universality of the mechanism of initiation of protein synthesis. Gene.

[28] Ding Y., Ren L. DNA genetic algorithm for design of the generalized membership-type takagi-sugeno fuzzy control system. Proceedings of the 2000 IEEE International Conference on Systems, Man and Cybernetics. Cybernetics Evolving to Systems, Humans, Organizations, and Their Complex Interactions.

[29] Zhang L., Wang N. (2013). A modified DNA genetic algorithm for parameter estimation of the 2-chlorophenol oxidation in supercritical water. Appl. Math. Model..

[30] Chen X., Wang N. (2010). Optimization of short-time gasoline blending scheduling problem with a DNA based hybrid genetic algorithm. Chem. Eng. Process. Process Intensif..

[31] Li Z., Ning W. (2013). An adaptive RNA genetic algorithm for modeling of proton exchange membrane fuel cells. Int. J. Hydrog. Energy.

[32] Zang W., Zhang W., Zhang W., Liu X. (2017). A genetic algorithm using triplet nucleotide encoding and DNA reproduction operations for unconstrained optimization problems. Algorithms.

[33] Amos M., Paun G., Rozenberg G., Salomaa A. (2002). Topics in the theory of DNA computing. Theor. Comput. Sci..

[34] Stanley K.O., Miikkulainen R. Efficient reinforcement learning through evolving neural network topologies. Proceedings of the Genetic & Evolutionary Computation Conference.

[35] Mattiussi C., Floreano D. Neuroevolution with analog genetic encoding. Proceedings of the International Conference on Parallel Problem Solving from Nature.

[36] Jiang F., Berry H., Schoenauer M. Unsupervised learning of echo state networks:balancing the double pole. Proceedings of the Conference on Genetic and Evolutionary Computation.

[37] Saravanan N., Fogel D.B. (2002). Evolving neural control systems. IEEE Expert.

[38] Wieland A.P. Evolving neural network controllers for unstable systems. Proceedings of the IJCNN-91-Seattle International Joint Conference on Neural Networks.

[39] Moriarty D.E., Miikkulainen R. (1996). Efficient reinforcement learning through symbiotic evolution. Mach. Learn..

[40] Gomez F.J., Miikkulainen R. Solving non-Markovian control tasks with neuro-evolution. Proceedings of the Sixteenth International Joint Conference on Artificial Intelligence.

[41] Whitley D., Pyeatt L. A comparison between cellular encoding and direct encoding for genetic neural networks. Proceedings of the Conference on Genetic Programming.

